# ^18^F-FDG-PET and Multimodal Biomarker Integration: A Powerful Tool for Alzheimer’s Disease Diagnosis

**DOI:** 10.1007/s13139-025-00932-2

**Published:** 2025-07-10

**Authors:** Yvonne Bouter, Robert M. Glasnek, Jannis M. Wenzel, Caroline Bouter

**Affiliations:** 1 Department of Psychiatry and Psychotherapy, University Medical Center Göttingen (UMG), Georg-August-University, Göttingen, Germany; 2 Department of Nuclear Medicine, University Medical Center Göttingen (UMG), Georg- August-University, Göttingen, Germany

**Keywords:** FDG-PET, Florbetapir, Alzheimer’s Disease, Positron Emission Tomography, Biomarker

## Abstract

An early, biomarker-based diagnosis of Alzheimer’s Disease (AD) is crucial, especially with the emerging availability of novel therapeutic options. However, the role of ^18^F-FDG-PET and its relationship to other PET and CSF biomarkers remains unclear. Therefore, the aim of this study was the evaluation of the role of ^18^F-FDG-PET in AD diagnosis and its relationship to other commonly used fluid and PET biomarkers and their individual and multimodal accuracy in AD diagnosis. We included *n* = 157 AD patients, *n* = 603 MCI patients, and *n* = 380 cognitively normal participants from the Alzheimer’s Disease Neuroimaging Initiative (ADNI) that underwent PET imaging with ^18^F-FDG or ^18^F-Florbetapir. Clinical and imaging data including patient characteristics, CSF biomarkers, cognition tests, ^18^F-FDG-PET, ^18^F-Florbetapir-PET, and ^18^F-Flortaucipir-PET were retrospectively analyzed. PET images were quantified in several brain regions. The uptake of ^18^F-FDG was inversely correlated with ^18^F-Florbetapir and positively correlated with CSF Aβ42 in several brain regions commonly affected by AD. Additionally, ^18^F-FDG uptake showed an inverse correlation with both forms of CSF tau, t-tau and p-tau, in various brain regions, but did not correlate with ^18^F-Flortaucipir uptake. Moreover, regional ^18^F-FDG uptake was positively correlated with cognitive function. Diagnostic accuracies were similarly high for ^18^F-FDG uptake in the PCC/Precuneus region, ^18^F-Florbetapir uptake, CSF Aβ42, CSF p-tau, and ^18^F-Flortaucipir uptake in differentiating AD from cognitively normal individuals. ^18^F-FDG-PET and its combination with CSF p-tau/ Aβ42 ratio showed the highest predictive power for disease severity. The study underscores the potential of integrating ^18^F-FDG-PET with CSF biomarkers to enhance the diagnosis, prognosis, and monitoring of AD, highlighting the complexity and regional specificity of biomarker interactions in neurodegeneration.

**Supplementary Information:**

The online version contains supplementary material available at 10.1007/s13139-025-00932-2.

## Introduction

Alzheimer’s disease (AD) is the most common form of dementia, with its prevalence steadily increasing due to an aging global population. To enable an accurate and early diagnosis, which is crucial for timely intervention in AD, reliable biomarkers are essential.

Biomarkers for AD can be categorized into three key groups based on the disease’s pathological hallmarks: amyloid biomarkers (A), tau biomarkers (T), and neurodegeneration biomarkers (N). The so-called A/T/N model as proposed by the “National Institute on Aging and Alzheimer’s Association” (NIA-AA) provides a framework for a biomarker-related definition of AD based on a patient’s individual biomarker profile. The model allows for a more comprehensive and individualized assessment of AD biomarkers in the diagnosis, prognosis, and monitoring of the disease [1].

Currently available biomarkers include fluid biomarkers for amyloid and tau, measured in cerebrospinal fluid (CSF) or plasma, as well as imaging biomarkers for amyloid, tau and neurodegeneration, using magnetic resonance imaging (MRI) or positron emission tomography (PET) [2–4]. Clinically, CSF biomarkers including amyloid-β 1–42 (Aβ42), total-tau (t-tau), and phosphorylated tau-^18^1 (p-tau) are commonly assessed [2].

In the clinical workup of AD patients CSF biomarkers amyloid-ß 1–42 (Aß42), total-tau (t-tau), and phosphorylated tau-^18^1 (p-tau) are commonly assessed. Among imaging biomarkers, ^18^F-Fluorodeoxyglucose (^18^F-FDG)-PET imaging is a clinically well-established neurodegeneration biomarker that is able to identify patterns of regional cortical hypometabolism. AD is typically characterized by a severe hypometabolism in the posterior cingulate cortex, the precuneus and the posterior temporoparietal cortex [5]. Furthermore, cortical amyloid-load can be assessed by amyloid-PET using different tracers that are able detect cortical amyloid deposition, including ^18^F-Florbetapir. Tau-PET has great potential in contributing to the accurate diagnosis of AD, but it is not yet established in the clinical AD assessment [6, 7].

With the emerging availability of amyloid-lowering monoclonal antibody therapies, biomarker-based diagnosis of AD has become even more critical [8–10]. Biomarker measurements of amyloid, tau, and neurodegeneration are integral to patient selection and efficacy monitoring in AD clinical trials [11]. Accurate biomarker profiles not only facilitate the early identification of suitable therapy candidates but also enable monitoring of disease progression, assessment of treatment effects, and prediction of conversion from mild cognitive impairment to AD.

However, the relationship of imaging and CSF biomarkers, especially with ^18^F-FDG-PET, remains unclear. Therefore, this study aims to address this gap by evaluating the impact of ^18^F-FDG-PET in the AD diagnosis and its relationship to other commonly used fluid and imaging biomarkers.

## Materials and Methods

### Study Participants

All study data were obtained from the Alzheimer’s Disease Neuroimaging Initiative (ADNI) database (http://adni.loni.usc.edu ); a multicentered project that unifies data on demographics, clinical and cognitive assessments as well as on genetic, biochemical and imaging biomarkers in AD, MCI, and CN subjects with the aim to improve clinical research on AD. Standardized protocols and unrestricted data access enable the analysis of an enormous volume of data from over 1800 participants included in the ADNI database to date. Groups of MCI patients, AD patients, and cognitively normal elderly controls were incorporated in four phases starting in 2004.

ADNI data from ADNI-1, ADNI-GO, ADNI-2, and ADNI-3, were downloaded from the ADNI database (see footnote 1) in May, 2023 (*n* = 1296). Participants without available ^18^F-Florbetapir or ^18^F-FDG PET in DICOM format were excluded (*n* = 156). Patient characteristics, Mini Mental State Examination (MMSE), Clinical Dementia Rating score (CDR), Alzheimer’s Disease Assessment Scale-Cognitive Subscale (ADAS- Cog), education years, ApoE4 status, CSF results (including Aβ42, t-tau and p-tau181), and PET imaging results (^18^F-Florbetapir, ^18^F-FDG PET and ^18^F-Flortaucipir) were assessed. Imaging and CSF data was not available for all patients; therefore, subset analysis was performed. Numbers of participants of each subset are summarized in supplementary Fig. 1.

Participants were categorized as cognitively normal, MCI, and AD following the ADNI protocol. AD patients exhibited notable subjective and objective memory impairment, as indicated by scores on the Wechsler Memory Scale - Revised (WMS-R) Logical Memory II subscale. This impairment affected their daily activities and was associated with a MMSE below 26 and a CDR of 0.5 or 1 and above. MCI patients were characterized by significant memory issues, with a MMSE ranging from 24 to 30 and a CDR of 0.5 or higher, along with an abnormal score on the WMS-R Logical Memory II subscale. Cognitively normal participants displayed no signs of dementia, demonstrating normal cognition with an MMSE ranging from 24 to 30, a CDR of 0, and a WMS-R Logical Memory II subscale score above education-adjusted cutoffs. Detailed information about the ADNI inclusion and exclusion criteria can be found online (see footnote 2). Three-group differentiation was used in all analyses.

### ^18^F-FDG-PET

The^18^F-FDG-PET imaging procedure adhered to a standardized imaging protocol with the injection of 185 (± 10%) MBq^18^F-FDG followed by a dynamic image acquisition of 6x5 minute frames 30-60 minutes post injection. Quality control was performed by the ADNI PET QC team, and all scans that passed the quality control were transmitted in DICOM format to the Laboratory of Neuroimaging (LONI) for storage. PET images underwent preprocessing including motion correction, time frame averaging, reorientation into a standardized 160 × 160 × 96 matrix with a voxel size of 1.5 mm, and smoothing with a scanner-specific filter function determined by the Hoffman phantom PET scans that were acquired during the certification process resulting in images of a uniform isotropic resolution of 8 mm full width at half maximum (for more details, refer to adni.loni.usc.edu).

^18^F-FDG-PET analysis was performed using the PMOD Neuro Tool (PMOD Technologies, Switzerland). Individual FDG-PET image files (DICOM) were loaded into the tool followed by MRI-template based spatial normalization using a SPM5 template as spatial reference standard. Alignment of the subjects individual PET and the template was evaluated and adjusted, if needed. PET images were co-registered to the single-subject brain atlas template AAL-Merged, which is the automatic anatomic labeling result of the spatially normalized single-subject T1-weighted MRI data set provided by the Montreal Neurological Institute. The atlas defines 71 cortical and non-cortical volumes of interest (VOI). VOI statistics (kBq/cc) were generated for the cortical VOI and standardized uptake values were calculated using the pons as a reference region (SUVR). 4 cortical regions that are mainly affected in AD were pooled and used for further analysis including: the frontal cortex, posterior cingulate cortex/precuneus, parietal cortex, and temporal cortex region.

### ^18^F-Florbetapir-PET Data

^18^F-Florbetapir-PET imaging was performed following the standard ADNI imaging protocol, involving the acquisition of 4 × 5-minute frames 50–70 min post-injection of 370 (± 10%) MBq ^18^F-Florbetapir. Quality control and preprocessing was performed as described above. PET image processing was performed with FreeSurfer v7.1.1 for an MRI-based definition of multiple cortical regions as well as reference regions for normalization as included in the ADNI UC Berkeley florbetapir dataset. SUVR were calculated using the cerebellum as reference region as. A cortical summary region, as well as 4 cortical regions (frontal, temporal, parietal cortex, and cingulate cortex/precuneus) were used for further analysis. Data were derived from the UCBERKELEYAV45_04_26_22.csv dataset, ADNI website).

### ^18^F-Flortaucipir-PET Data

^18^F-Flortaucipir-PET was performed after a standard protocol administering 370 (± 10%) MBq ^18^F- Flortaucipir with an image acquisition 75–105 min post-injection with 6 × 5 min frames. was Quality control and preprocessing was performed as described above. Further PET image processing was performed with FreeSurfer v7.1.1 for an MRI-based definition of multiple cortical regions as well as reference regions for normalization. The inferior cerebellum was used as reference region and SUVRs of frontal, parietal, temporal, and cingulate cortex/precuneus regions were obtained as described in the ADNI Tau PET Processing Methods protocol^1^. Data were derived from the UCBERKELEY_TAU_6MM.csv dataset, ADNI website).

### Statistical Analyses

Statistical analyses were conducted with SPSS Statistics version 27 (IBM, Armonk, NY, United States) and GraphPad Prism version 9 (GraphPad Software, San Diego, CA, United States). Differences between groups were assessed using the Kruskal–Wallis test. Univariate analysis of covariance (ANCOVA) was applied for relevant covariate adjustment, as indicated. Where appropriate, post-hoc comparisons were adjusted using Bonferroni correction to account for multiple comparisons. For categorical variables, the Chi-square test or Fisher’s exact test were utilized. Relationships between two variables were explored using Spearman correlation and simple linear regression across a maximum of four predefined brain regions. No formal correction for multiple comparisons was applied given the limited number of regions tested. The majority of correlations showed highly significant p-values (*p* < 0.001), supporting the robustness of the findings. To evaluate diagnostic accuracy, Receiver Operating Characteristic (ROC) analysis was performed. Binary logistic regression analysis was performed to evaluate the diagnostic accuracy in distinguishing between diagnoses. To combine multiple variables, performance of the model was assessed using predicted probabilities and the score was used for further ROC-analysis. Furthermore, multiple linear regression was used to evaluate predictive power of individual biomarkers and their combinations. Significance levels were denoted as follows: ∗*p* < 0.05; ∗∗*p* < 0.01; ∗∗∗*p* < 0.001.

## Results

### Baseline Patient Characteristics

A total of *n* = 1140 participants with available 18 F-FDG PET or 18 F-Florbetapir PET scans were included in the study (Supplementary Fig. 1). *N* = 157 participants had a diagnosis of AD, *n* = 603 MCI, and *n* = 380 were cognitively normal. Baseline characteristics are shown in Table 1.

**Table 1:** Patient’s characteristics. AD = Alzheimer’s disease; mci = mild cognitive impairment; CN = cognitive normal subjects; mmse = mini mental state examination; cdr = clinical dementia rating; ADAS-Cog = Alzheimer’s disease assessment Scale-Cognitive subscale

*N* = 1140	AD (*n* = 157)	MCI (*n* = 603)	CN (*n* = 380)	*p*-value
Age (years; mean ± SD)	74.28 (± 8.441)	74.35 (± 7.680)	74.86 (± 7.061)	0.7158
MMSE (mean ± SD)	22.29 (± 3.175)	27.01 (± 3.240)	28.90 (± 1.474)	< 0.0001
CDR (mean ± SD)	0.8408 (± 0.3351)	0.5328 (± 0.3087)	0.000 (± 0.000)	< 0.0001
ADAS-Cog (mean ± SD)	31.33 (± 9.125)	16.47 (± 9.515)	9.287 (± 5.187)	< 0.0001
Education (years; mean ± SD)	15.69 (± 2.623)	16.11 (± 2.717)	16.64 (± 2.550)	0.0004
Gender				0.0009
Female	64	256	203	
Male	93	347	177	
ApoE4 Genotype				< 0.0001
ε2/ε2	1	-	1	
ε2/ε3	6	39	41	
ε3/ε3	46	270	226	
ε2/ε4	2	14	6	
ε3/ε4	70	214	93	
ε4/ε4	32	66	13	

### ^18^F-FDG-PET

**Fig. 1:**
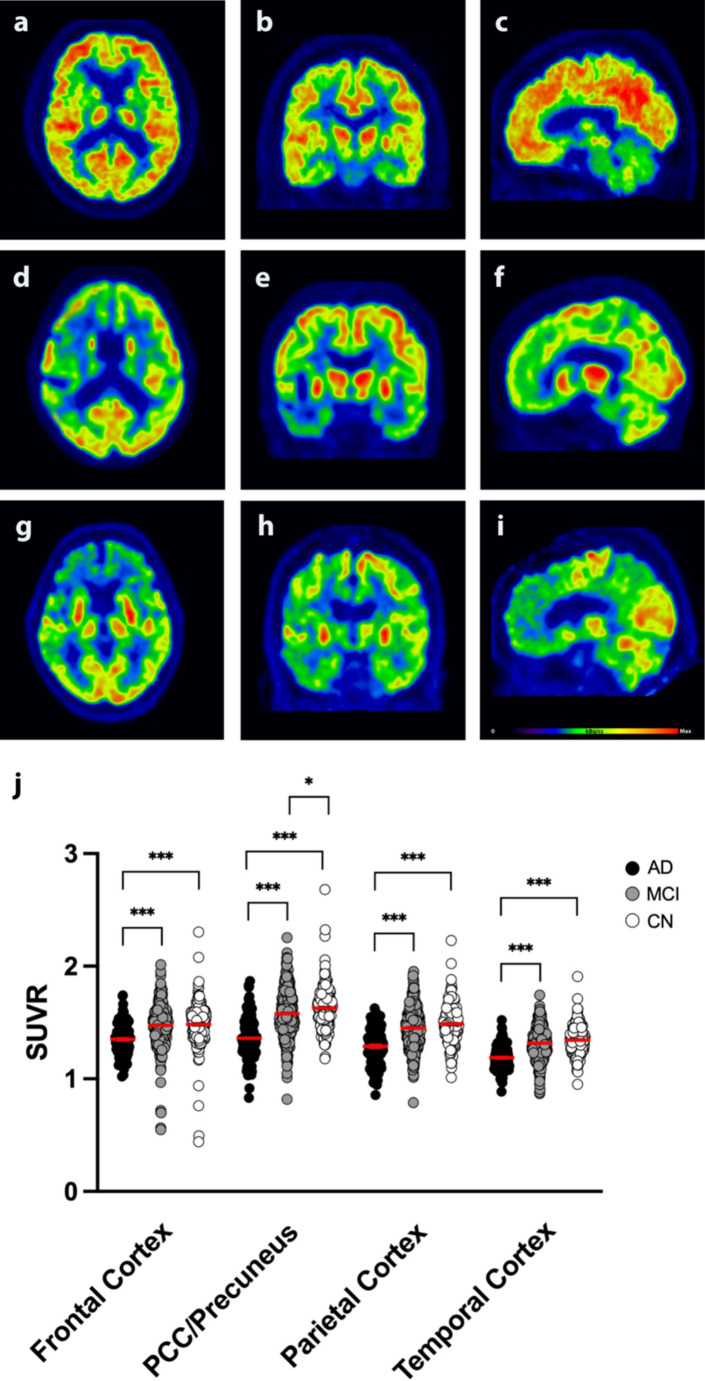
^18^F-FDG-PET. (**a-c**) Representative ^18^F-FDG-PET images of a cognitively normal subject in transverse (**a**), coronal (**b**), and sagittal (**c**) planes. (**d-f**) ^18^F-FDG-PET images of an MCI patient in transverse (**d**), coronal (**e**), and sagittal (**f**) planes. (**g-h**) ^18^F-FDG-PET images of an AD patient in transverse (**g**), coronal (**h**), and sagittal (**i**) planes. (**j**) SUVR was significantly lower in AD patients compared to MCI and CN patients in the frontal cortex, posterior cingulate cortex/precuneus, parietal cortex, and temporal cortex regions. ANCOVA with post-hoc Bonferroni correction; *p < 0.05; ***p < 0.0001; *n* = 752. AD = Alzheimer’s dementia; MCI = Mild Cognitive Impairment; CN = Cognitive Normal; PCC = Posterior Cingulate Cortex; SUVR = Standardized Uptake Value Ratio

^18^F-FDG-PET scans were available in *n* = 752 participants (AD: *n* = 99; MCI: *n* = 449; CN: *n* = 204). ^18^F-FDG uptake was significantly lower in the frontal cortex, posterior cingulate cortex/precuneus, parietal cortex, and temporal cortex regions in AD patients compared to MCI and CN participants whilst adjusting for gender as a covariate (*p* < 0.0001 in all regions; ANCOVA). ^18^F-FDG uptake was lower in the PCC/Precuneus region in MCI patients compared to CN subjects whilst adjusting for gender as a covariate (*p* = 0.0123; ANCOVA; Fig. 1).

### Diagnostic Accuracy of ^18^F-FDG-PET

**Fig. 2:**
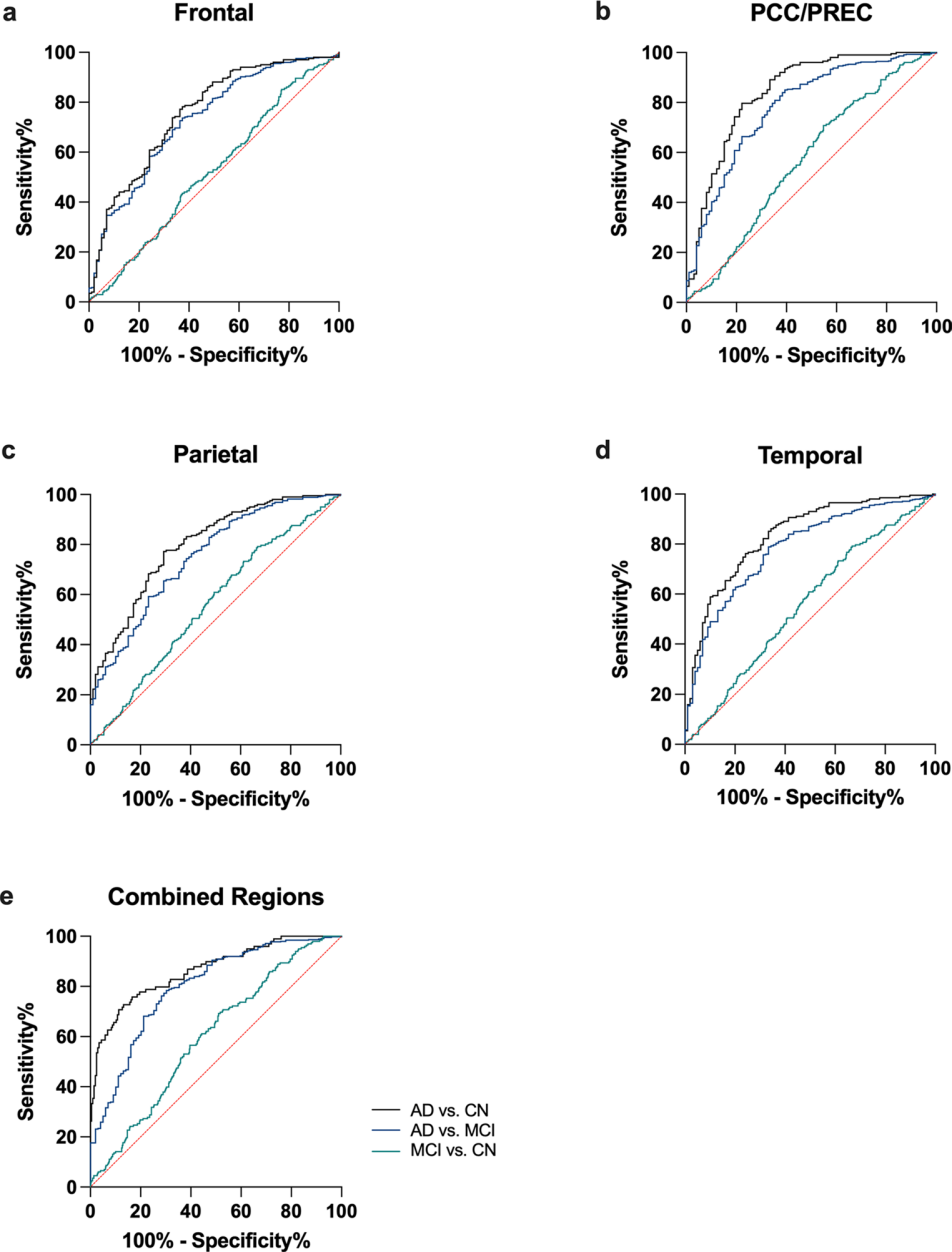
^18^F-FDG-PET Receiver-operating characteristic analysis. (**a-e**) Diagnostic accuracy of hypometabolism was the highest for differentiating AD from CN in all tested brain regions and the combination of all 4 regions (**e**). The dotted red line shows the diagonal reference. AD = Alzheimer’s dementia; MCI = Mild Cognitive Impairment; CN = Cognitive Normal

Detailed results of ROC analyses can be found in Table 2. The ^18^F-FDG uptake in the PCC/Precuneus and the temporal cortex showed the highest diagnostic accuracy to differentiate between AD and CN (PCC/Precuneus: AUC = 0.843; 95% CI: 0.7927 to 0.8946; temporal cortex: AUC: 0.8345; 95% CI: 0.7857 to 0.8833; Fig. 2). Combining all four brain regions lead to an improvement of diagnostic accuracy (AUC: 0.8637; 95% CI: 0.8183 to 0.9091; ROC analysis). The highest diagnostic accuracy to differentiate between AD and MCI was also achieved in the PCC/Precuneus (AUC = 0.7833; 95% CI: 0.7314 to 0.8353) and the temporal cortex (AUC: 0.783; 95% CI: 0.7345 to 0.8314), while combining all four brain regions lead to an improvement of diagnostic accuracy (AUC: 0.7965; 95% CI: 0.7472 to 0.8459; ROC analysis). The diagnostic accuracy to differentiate between MCI and CN was low in all regions (frontal cortex: AUC = 0.5220; 95% CI: 0.4751 to 0.5689; PCC/Precuneus: AUC = 0.5717; 95% CI: 0.5263 to 0.6172; parietal cortex: AUC = 0.5591; 95% CI: 0.5123 to 0.6059; temporal cortex: AUC = 0.5583; 95% CI: 0.5116 to 0.6051). Diagnostic accuracy of the combination of all four brain regions was improved, but still low (AUC: 0.5988; 95% CI: 0.5533 to 0.6444; ROC analysis).

**Table 2:** Diagnostic accuracy of biomarkers and their combination with ^18^F-FDG-PET. AD = Alzheimer’s disease; mci = mild cognitive impairment; CN = cognitive normal subjects; pcc = posterior cingulate cortex;

	AD and CN		AD and MCI		MCI and CN	
	AUC	95% CI	AUC	95% CI	AUC	95% CI
FDG-PET (*n* = 752)						
frontal	0.7571	0.6984 to 0.8158	0.7305	0.6758 to 0.7852	0.5220	0.4751 to 0.5689
parietal	0.7978	0.7451 to 0.8505	0.7496	0.6967 to 0.8026	0.5591	0.5123 to 0.6059
PCC/Precuneus	0.843	0.7927 to 0.8946	0.7833	0.7314 to 0.8353	0.5717	0.5263 to 0.6172
temporal	0.8345	0.7857 to 0.8833	0.783	0.7345 to 0.8314	0.5583	0.5116 to 0.6051
All regions	0.8637	0.8183 to 0.9091	0.7965	0.7472 to 0.8459	0.5988	0.5533 to 0.6444
^18^ F-Florbetapir-PET (*n* = 1119)						
Whole brain	0.8265	0.7823 to 0.8707	0.7107	0.6643 to 0.7571	0.6279	0.5929 to 0.6630
^18^F-Florbetapir-PET + ^18^F-FDG-PET	0.9055	0.8670 to 0.9441	0.8133	0.7657 to 0.8608	0.6611	0.6179 to 0.7043
CSF (*n* = 750)						
Aβ42	0.8727	0.8378 to 0.9076	0.7289	0.6830 to 0.7748	0.7080	0.6716 to 0.7445
Aβ42 + FDG	0.912	0.874 to 0.95	0.834	0.789 to 0.88	0.6^18^	0.57 to 0.666
t-tau	0.5656	0.5129 to 0.6183	0.6490	0.6036 to 0.6945	0.5624	0.5209 to 0.6038
t-tau + FDG	0.883	0.839 to 0.927	0.82	0.772 to 0.869	0.594	0.544 to 0.645
p-tau	0.8529	0.8163 to 0.8895	0.6996	0.6544 to 0.7447	0.6591	0.6220 to 0.6962
p-tau + FDG	0.936	0.906 to 0.967	0.839	0.796 to 0.883	0.637	0.59 to 0.684
p-tau/Aβ42 Ratio	0.8403	0.7787 to 0.9020	0.7051	0.6432 to 0.7670	0.6206	0.5666 to 0.6746
p-tau/Aβ42 Ratio + FDG	0.944	0.913 to 0.974	0.846	0.802 to 0.89	0.65	0.603 to 0.696
^18^ F-Flortaucipir-PET (*n* = 399)						
frontal	0.7291	0.5314 to 0.9269	0.6385	0.4530 to 0.8241	0.5863	0.5232 to 0.6495
parietal	0.8405	0.6579 to 1	0.7504	0.5753 to 0.9255	0.5821	0.5184 to 0.6457
PCC/Precuneus	0.8551	0.7068 to 1	0.7608	0.6073 to 0.9143	0.6015	0.5398 to 0.6631
temporal	0.8370	0.6450 to 1	0.7239	0.5459 to 0.9019	0.6224	0.5639 to 0.6808
^18^F-Flortaucipir-PET + ^18^F-FDG-PET	na	na	na	na	0.63	0.551 to 0.71

### ^18^F-FDG Uptake and Cognition

^18^F-FDG uptake correlated with both MMSE and ADAS-Cog in all tested brain regions (MMSE: Frontal: *p* < 0.0001; *r* = 0.3384; PCC/Precuneus: *p* < 0.0001; *r* = 0.4322; Parietal: *p* < 0.0001; *r* = 0.3587; Temporal: *p* < 0.0001; *r* = 0.4243; ADAS-Cog: Frontal: *p* < 0.0001; *r*= −0.3447; PCC/Precuneus: *p* < 0.0001; *r*= −0.4654; Parietal: *p* < 0.0001; *r*= −0.4131; Temporal: *p* < 0.0001; *r*= −0.4618; Spearman correlation).

### Amyloid-PET with ^18^F-Florbetapir

**Fig. 3:**
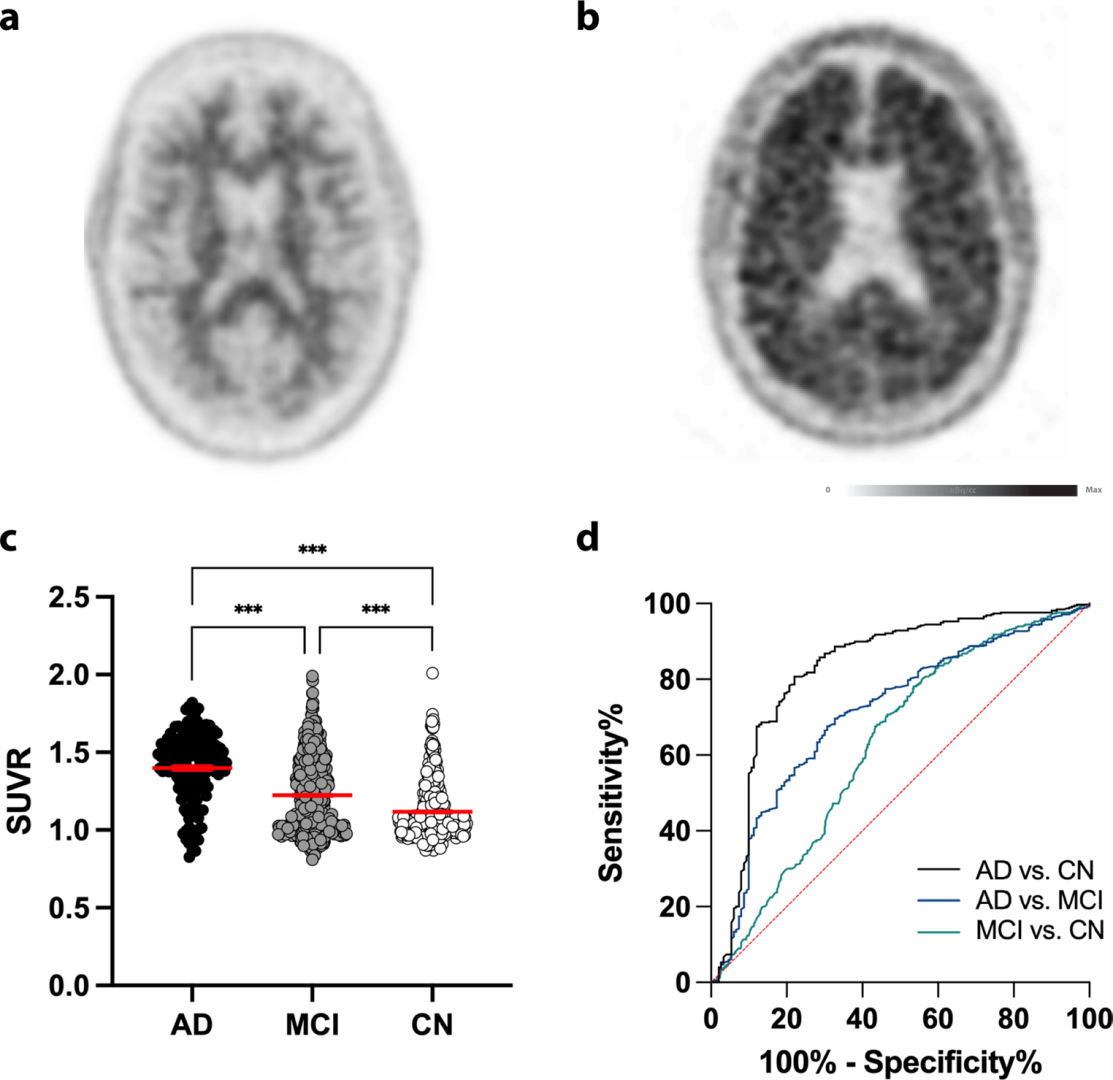
^18^F-Florbetapir-PET results. (**a-b**) Representative ^18^F-Florbetapir-PET images: (**a**) Scan showing no significant amyloid load and (**b**) scan showing increased amyloid load. (**c**) SUVR was significantly higher in AD patients compared to MCI and CN patients in the whole brain region (*n* = 1119). (**d**) Receiver-operating characteristic analysis. Diagnostic accuracy was the highest for differentiating AD from CN. The dotted red line shows the diagonal reference. AD = Alzheimer’s dementia; MCI = Mild Cognitive Impairment; CN = Cognitive Normal; SUVR = Standardized Uptake Value Ratio. ANCOVA with post-hoc Bonferroni correction; ***p < 0.0001

^18^F-Florbetapir scans were available in *n* = 1119 participants (AD: *n* = 150; MCI: *n* = 590; CN: *n* = 379). SUVR was significantly higher in AD patients compared to MCI and CN subjects whilst adjusting for gender as a covariate (*p* < 0.0001; ANCOVA). Furthermore, SUVR was significantly higher in MCI patients compared to CN subjects whilst adjusting for gender as a covariate (*p* < 0.0001; ANCOVA; Fig. 3). ^18^F-Florbetapir uptake was significantly higher in the PCC/Precuneus region compared to all other tested brain regions in AD, MCI and CN (*p* < 0.0001; ANCOVA).

### Diagnostic Accuracy of ^18^F-Florbetapir

ROC analysis yielded an AUC of 0.8265 with a 95% confidence interval of 0.7823 to 0.8707 of ^18^F-Florbetapir whole brain uptake to differentiate between AD and CN (Table 2; Fig. 3d). AUC of ^18^F-Florbetapir whole-brain uptake in distinguishing between AD and MCI was 0.7107, with a 95% confidence interval ranging from 0.6643 to 0.7571 and in MCI and CN 0.6279, with a 95% confidence interval ranging from 0.5929 to 0.6630. The combination of FDG- and ^18^F-Florbetapir-PET results showed an improvement of diagnostic accuracy (AD vs. CN: AUC: 0.9055; 95% CI: 0.8670 to 0.9441 CNAD; AD vs. MCI: 0.8133; 95% CI: 0.7657 to 0.8608; MCI vs. CN: 0.6611; 95% CI: 0.6179 to 0.7043).

### ^18^F-Florbetapir Uptake and Cognition

^18^F-Florbetapir uptake in the whole brain correlated with both cognition tests, MMSE (*p* < 0.001; *r*= −0.4305) and ADAS-Cog (*p* < 0.001; *r* = 0.4295; Spearman correlation).

### Correlation of Regional Amyloid Load and ^18^F-FDG Uptake

A total of *n* = 732 participants underwent PET imaging using both tracers, 18F-FDG and 18F-Florbetapir. Regional ^18^F-Florbetapir uptake showed an inverse correlation to ^18^F-FDG uptake in all tested cortical regions (Frontal: *p* < 0.0001; *r*= −0.^18^08; PCC/Precuneus: *p* < 0.0001; *r*= −0.3114; Parietal: *p* < 0.0001; *r*= −0.2144; Temporal: *p* < 0.0001; *r*= −0.2768; Spearman correlation).

**Fig. 4:**
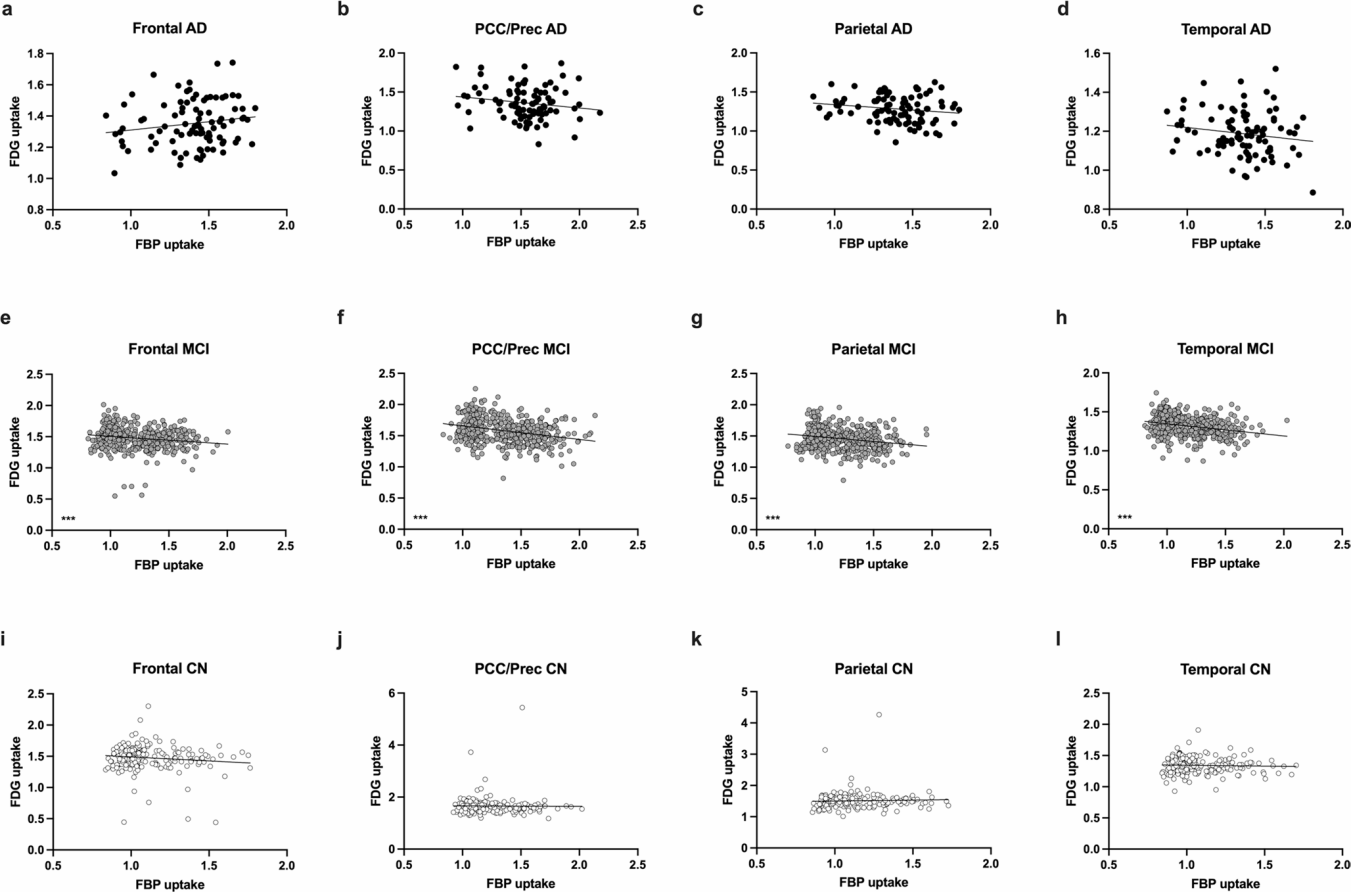
Correlation of regional Amyloid load and ^18^F-FDG uptake. Regional ^18^F-Florbetapir (FBP) uptake correlated inversely with ^18^F-FDG uptake in MCI patients in all tested brain regions (**e-h**), but not in AD (**a-d**) and CN (**i-l**) patients (*p* > 0.1; Spearman correlation; *n* = 732). ***p < 0.0001. AD = Alzheimer’s dementia; MCI = Mild Cognitive Impairment; CN = Cognitive Normal; PCC/Prec = Posterior Cingulate Cortex and Precuneus

While regional ^18^F-Florbetapir uptake correlated negatively in all regions in the subgroup of MCI patients (Frontal: *p* < 0.0001; *r*= −0.^18^38; PCC/Precuneus: *p* < 0.0001; *r*= −0.2838; Parietal: *p* < 0.0001; *r*= −0.2127; Temporal: *p* < 0.0001; *r*= −0.2712; Spearman correlation), it did not correlate with ^18^F-FDG uptake in the subgroups of AD patients and CN controls in all tested cortical regions (AD: Frontal: *p* = 0.1950; *r* = 0.1371; PCC/Precuneus: *p* = 0.1667; *r*= −0.1462; Parietal: *p* = 0.1029; *r*= −0.1720; Temporal: *p* = 0.2075; *r*= −0.1334; CN: Frontal: *p* = 0.86; *r*= −0.01272; PCC/Precuneus: *p* = 0.2552; *r*= −0.0802; Parietal: *p* = 0.2139; *r* = 0.0876; Temporal: *p* = 0.9267; *r* = 0.0065; Spearman correlation; Fig. 4).

### Cerebrospinal Fluid

CSF measurements of Aβ42, t-tau and p-tau were available in *n* = 750 participants (AD: *n* = 132; MCI: *n* = 437; CN: *n* = 181). Mean CSF biomarker results are shown in Table 3. CSF Aβ42 was significantly lower in AD patients compared to MCI and CN participants whilst adjusting for gender as a covariate (*p* < 0.0001; ANCOVA). MCI patients also showed lower CSF Aβ42 values compared to CN subjects (*p* = 0.0033; ANCOVA).

**Table 3:** CSF results. AD = Alzheimer’s disease; mci = mild cognitive impairment; CN = cognitive normal subjects

*N* = 750	AD	MCI	CN	*p*-value
CSF				
Abeta42 (pg/ml; mean ± SD)	179.7 (± 63.64)	228.2 (± 80.40)	249.5 (± 77.84)	< 0.0001
t-tau (pg/ml; mean ± SD)	118.8 (± 56.46)	81.28 (± 46.60)	64.19 (± 27.47)	< 0.0001
p-tau (pg/ml; mean ± SD)	34.18 (± 17.77)	26.53 (± 13.92)	21.92 (± 9.09)	< 0.0001
p-tau/Abeta42 Ratio	0.2840 (± 0.18)	0.1802 (± 0.13)	0.1255 (± 0.09)	< 0.0001

Both, t-tau and p-tau values were significantly higher in AD patients compared to MCI and CN whilst adjusting for gender as a covariate (*p* < 0.0001; ANCOVA). MCI patients also showed higher CSF t-tau and p-tau values compared to CN (t-tau: *p* = 0.0009; p-tau: *p* = 0.0011; ANCOVA).

Furthermore, the p-tau/Aβ42 ratio was significantly higher in AD patients compared to MCI and CN whilst adjusting for gender as a covariate (*p* < 0.0001; ANCOVA).

### Diagnostic Accuracy of CSF Biomarkers

The diagnostic accuracy of CSF Aβ42 was the highest in differentiating AD from CN (AUC: 0.8727, 95% CI: 0.8378 to 0.9076; Table 2), while a combination of FDG- and CSF Aβ42 results showed a further improvement of diagnostic accuracy (0.912; 95% CI: 0.874 to 0.95). The diagnostic accuracy of t-tau was low in differentiating between AD and CN (AUC = 0.5656; 95% CI: 0.5129 to 0.6183), as well as in AD and MCI (AUC = 0.6490; 95% CI: 0.6036 to 0.6945), and MCI and CN (AUC = 0.5624; 95% CI: 0.5209 to 0.6038; ROC analysis), while a combination of FDG- and t-tau results showed a slight improvement of diagnostic accuracy (Table 2).Using CSF p-tau, the highest diagnostic accuracy was achieved for the differentiation of AD from CN (AUC: 0.8529; 95% CI: 0.8163 to 0.8895), which was further improved by the combination with FDG (AUC: 0.936; 95% CI: 0.906 to 0.967; Table 2). The diagnostic accuracy of p-tau/Aβ42 Ratio was also the highest in differentiating AD from CN (AUC: 0.8403; 95% CI: 0.7787 to 0.9020; Table 2). A combination of FDG- and the p-tau/Aβ42 Ratio showed the best diagnostic accuracy overall (AUC: 0.944; 95% CI: 0.913 to 0.974).

### CSF and Cognition

CSF Aβ42 correlated with MMSE scores (*p* < 0.001; *r* = 0.4305), while CSF t-tau and p-tau showed an inverse correlation with MMSE (t-tau: *p* < 0.001; −0.4172; p-tau: *p* < 0.001; *r*= −0.3714). Furthermore, CSF Aβ42 showed an inverse correlation with ADAS-Cog (*p* < 0.001; *r*= −0.4333). CSF t-tau and p-tau correlated with ADAS-Cog (t-tau: *p* < 0.001; 0.4437; p-tau: *p* < 0.001; 0.3995, Spearman correlation).

### Correlation between ^18^F-FDG-PET and CSF Biomarkers

**Fig. 5:**
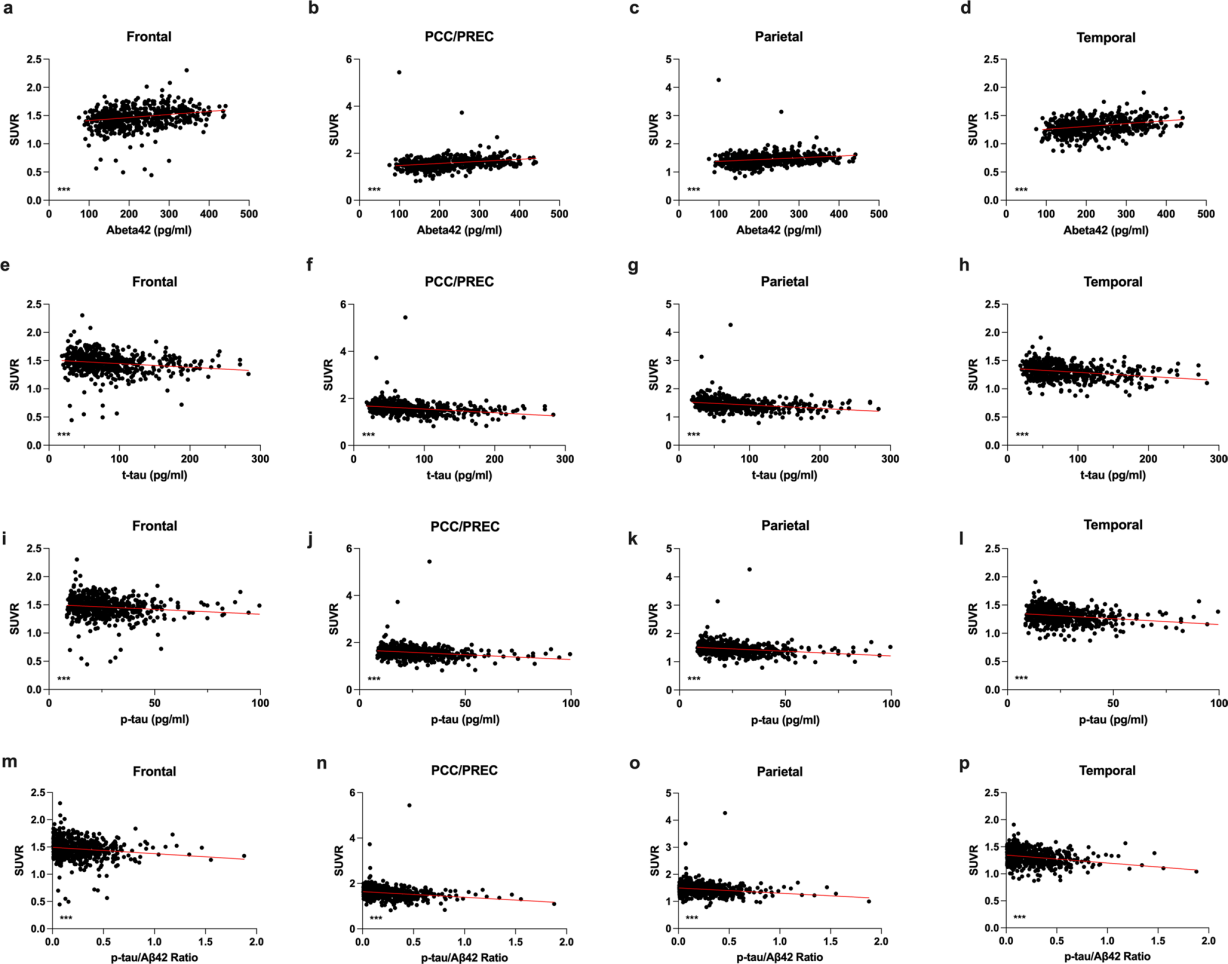
Correlation of regional ^18^F-FDG uptake and CSF biomarkers. Regional 18F-FDG uptake correlated with Aβ42 in all tested brain regions (**a-d**). 18F-FDG uptake correlated inversely with t-tau (**e-h**) and p-tau (**i-l**) in all tested brain regions (Spearman correlation; *n* = 545). ***p < 0.0001. PCC/Prec = Posterior Cingulate Cortex and Precuneus; SUVR = Standardized Uptake Value Ratio

Lumbar puncture was performed in *n* = 545 participants with available 18F-FDG PET scans. CSF Aβ42 correlated with ^18^F-FDG uptake in all tested cortical regions (Frontal: *p* < 0.0001; *r* = 0.2781; PCC/Precuneus: *p* < 0.0001; *r* = 0.3533; Parietal: *p* < 0.0001; *r* = 0.2964; Temporal: *p* < 0.0001; *r* = 0.3382; Spearman correlation; Fig. 8).CSF t-tau and p-tau correlated negatively with ^18^F-FDG uptake in all tested cortical regions (t-tau: Frontal: *p* < 0.0001; *r*= −0.2248; PCC/Precuneus: *p* < 0.0001; *r*= −0.3626; Parietal: *p* < 0.0001; *r*= −0.3450; Temporal: *p* < 0.0001; *r*= −0.2624; p-tau: Frontal: *p* < 0.0001; *r*= −0.1791; PCC/Precuneus: *p* < 0.0001; *r*= −0.3080; Parietal: *p* < 0.0001; *r*= −0.2987; Temporal: *p* < 0.0001; *r*= −0.2336; Spearman correlation). The p-tau/Aβ42 Ratio correlated negatively with ^18^F-FDG uptake in all tested cortical regions (Frontal: *p* < 0.0001; *r*=−0.2637; PCC/Precuneus: *p* < 0.0001; *r*=−0.2795; Parietal: *p* < 0.0001; *r*= −0.2578; Temporal: *p* < 0.0001; *r*=−0.2483; Spearman correlation; Fig. 5).

### ^18^F-Flortaucipir-PET

**Fig. 6:**
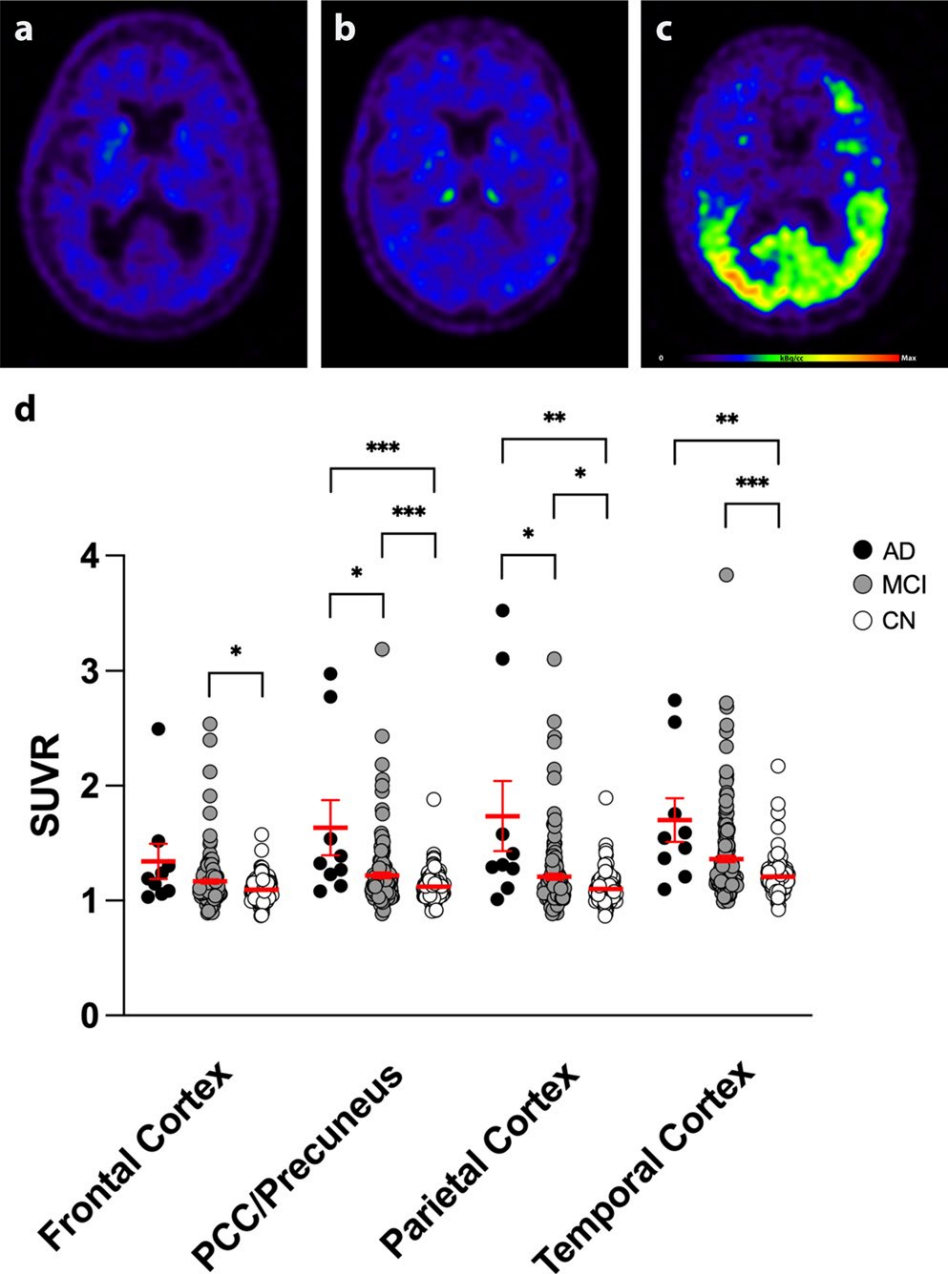
^18^F-Flortaucipir-PET results. (**a-c**) Representative ^18^F-Flortaucipir-PET images of a cognitively normal subject (**a**), an MCI patient (**b**), and an AD patient (**c**) in transverse plane. SUVR was significantly higher in AD patients compared to CN in in the posterior cingulate cortex/precuneus (*p* *<* 0.001), parietal (p = 0.0018), and temporal cortex (*p =* 0.002) as well as in the posterior cingulate cortex/precuneus (*p =* 0.03) and parietal cortex (*p =* 0.0307) compared to MCI. SUVR was significantly higher in MCI patients compared to CN in all tested brain regions (frontal cortex: *p =* 0.0173, posterior cingulate cortex/precuneus: *p =* 0.0036, parietal cortex: *p =* 0.0258, and temporal cortex: *p < *0.0001; *n =* 399). ANCOVA with post-hoc Bonferroni correction; *p < 0.05; **p < 0.01; ***p < 0.0001. SUVR = Standardized Uptake Value Ratio

^18^F-Flortaucipir-PET scans were available in *n* = 399 participants (AD: *n* = 9; MCI: *n* = 165; CN: *n* = 225). ^18^F-Flortaucipir uptake was significantly higher in AD patients compared to CN subjects in in the posterior cingulate cortex/precuneus (*p* < 0.001), parietal (*p* = 0.0018) and temporal cortex (*p* = 0.002; ANCOVA), whilst adjusting for gender as a covariate. ^18^F-Flortaucipir uptake was significantly higher in AD patients compared to MCI in the posterior cingulate cortex/precuneus (*p* = 0.03) and parietal cortex (*p* = 0.0307; ANCOVA), whilst adjusting for gender as a covariate. ^18^F-Flortaucipir uptake was significantly higher in MCI patients compared to CN in all tested brain regions whilst adjusting for gender as a covariate (frontal cortex: *p* = 0.0173, posterior cingulate cortex/precuneus: *p* = 0.0036, parietal cortex: *p* = 0.0258, and temporal cortex: *p* < 0.0001; ANCOVA; Fig. 6).

### Diagnostic Accuracy − ^18^F-Flortaucipir-PET

The ^18^F-Flortaucipir uptake in the PCC/Precuneus, parietal cortex, and the temporal cortex showed the highest diagnostic accuracy to differentiate between AD and CN (PCC/Precuneus: AUC = 0.8551; 95% CI: 0.7068 to 1; parietal cortex: AUC = 0.8405; 95% CI: 0.6579 to 1; temporal cortex: AUC: 0.8370; 95% CI: 0.6450 to 1; Table 2). The highest diagnostic accuracy between AD and MCI was detected in the PCC/Precuneus (AUC = 0.7608; 95% CI: 0.6073 to 0.9143). The diagnostic accuracy to differentiate between MCI and CN was low in all regions (frontal cortex: AUC = 0.5863; 95% CI: 0.5232 to 0.6495; PCC/Precuneus: AUC = 0.6015; 95% CI: 0.5398 to 0.6631; parietal cortex: AUC = 0.5821; 95% CI: 0.5184 to 0.6457; temporal cortex: AUC = 0.6224; 95% CI: 0.5639 to 0.6808; ROC analysis).

### ^18^F-Flortaucipir and Cognition

^18^F-Flortaucipir uptake correlated with the ADAS-Cog score in all tested brain regions (Frontal: *p* < 0.001; *r* = 0.2244; PCC/Precuneus: *p* < 0.001; *r* = 0.2350; Parietal: *p* < 0.001; *r* = 0.2457; Temporal: *p* < 0.001; *r* = 0.3029). Furthermore, ^18^F-Flortaucipir uptake showed an inverse correlation to MMSE in all regions (Frontal: *p* = 0.002; *r*= −0.1704; PCC/Precuneus: *p* < 0.001; *r*= −0.1837; Parietal: *p* < 0.001; *r*= −0.2548; Temporal: *p* < 0.001; *r*= −0.2587, Spearman correlation).

### Correlation of Regional ^18^F-Flortaucipir and ^18^F-FDG Uptake

**Fig. 7:**
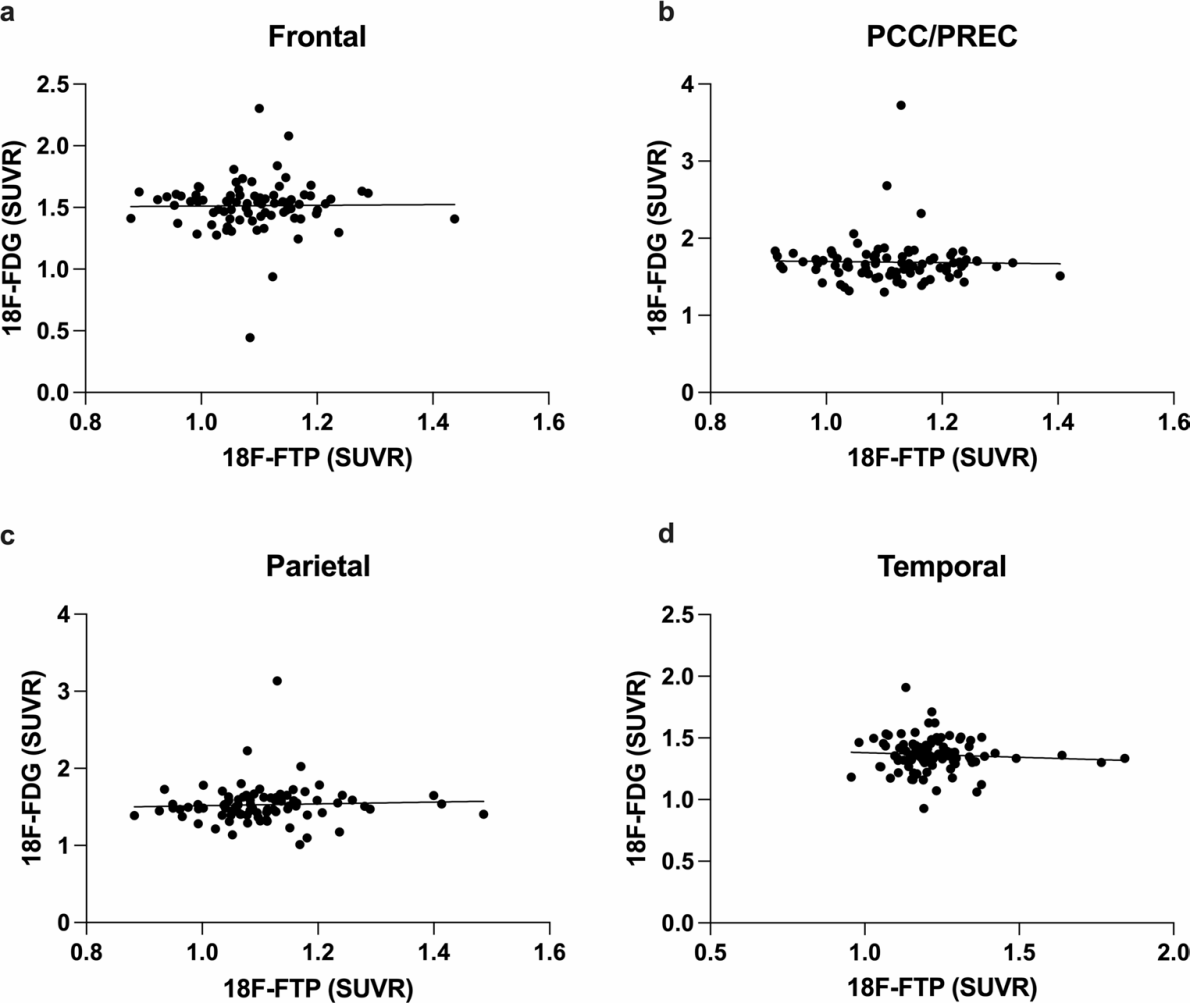
Correlation of regional 18F-Flortaucipir uptake and ^18^F-FDG uptake. (**A-D**) _18_F-Flortaucipir did not correlate significantly with ^18^F-FDG uptake in all tested cortical regions (*p > *0.23 in all regions; Spearman correlation; *n =* 219). SUVR = Standardized Uptake Value Ratio

PET scans using both ^18^F-Flortaucipir and 18F-FDG were available in *n* = 219 participants. Regional ^18^F-Flortaucipir uptake did not correlate with ^18^F-FDG uptake in any of the tested cortical regions (Frontal: *p* = 0.9028; *r* = 0.0135; PCC/Precuneus: *p* = 0.52; *r*= −0.0712; Parietal: *p* = 0.2377; *r* = 0.1302; Temporal: *p* = 0.6275; *r*= −0.05041; Spearman correlation; Fig. 7).

### Correlation of Regional ^18^F-Flortaucipir and ^18^F-Florbetapir Uptake

**Fig. 8:**
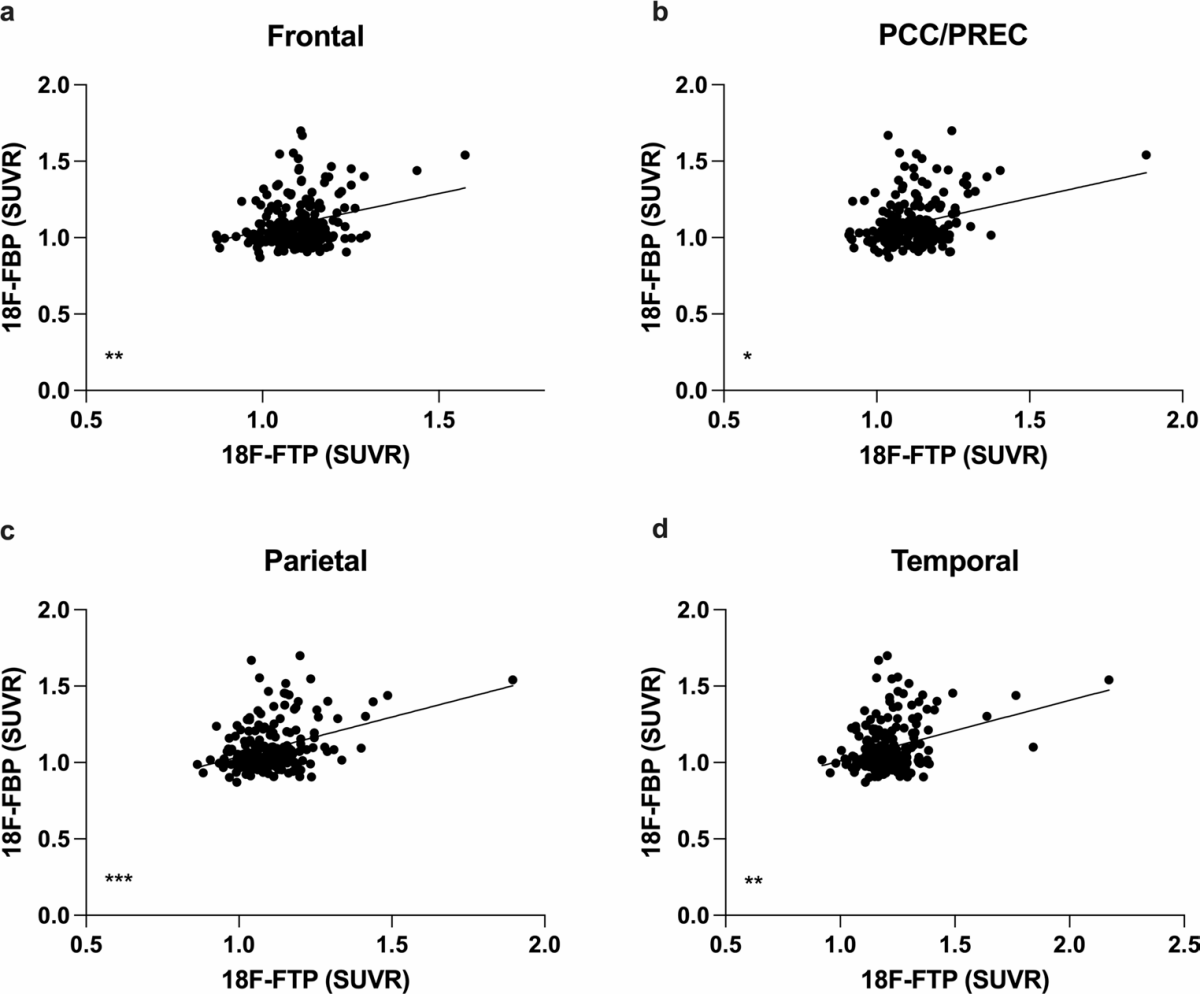
Correlation of regional ^18^F-Flortaucipir uptake and Amyloid load. (**a-d**) ^18^F-Flortaucipir correlated significantly with ^18^F- Florbetapir uptake in all tested regions (*p <* 0.02; Spearman correlation; *n = *399). SUVR = Standardized Uptake Value Ratio

PET imaging with ^18^F-Flortaucipir and ^18^F-Florbetapir was conducted in *n* = 399 participants. Regional ^18^F- Flortaucipir uptake correlated with ^18^F-Florbetapir uptake in all tested cortical regions (Frontal cortex: *p* = 0.0062; *r* = 0.^18^86; PCC/Precuneus: *p* = 0.0166; *r* = 0.1656; Parietal cortex: *p* < 0.0001; *r* = 0.2802; Temporal cortex: *p* = 0.0036; *r* = 0.1931; Spearman correlation; Fig. 8).

### Correlation of Regional ^18^F-Flortaucipir Uptake and CSF Biomarkers

*N* = 346 participants with an available ^18^F-Flortaucipir-PET underwent lumbar puncture. ^18^F-Flortaucipir uptake correlated with CSF Aβ42 in the posterior cingulate cortex/precuneus (*P* = 0.0226; *r*= −0.2939) and the temporal cortex (*p* = 0.0256; *r*= −0.2748; Spearman correlation). However, CSF tau markers did not correlate with cortical ^18^F-Flortaucipir uptake (t-tau: Frontal cortex: *p* = 0.3038; *r* = 0.1374; PCC/Precuneus: *p* = 0.8092; *r* = 0.0324; Parietal cortex: *p* = 0.8716; *r* = 0.0217; Temporal cortex: *p* = 0.4777; *r* = 0.09034; p-tau: Frontal cortex: *p* = 0.6742; *r* = 0.0554; PCC/Precuneus: *p* = 0.4879; *r*= −0.0913; Parietal cortex: *p* = 0.4359; *r*= −0.1025; Temporal cortex: *p* = 0.8817; *r*= −0.0187; Spearman correlation).

### Predictive Power of Various Biomarkers on Disease Severity

For the evaluation of the accuracy of the prediction of disease severity, using ADAS-Cog scores as a marker for cognitive decline, we applied multiple linear regression models (Table 4). ^18^F-FDG uptake including all four analyzed regions provided the strongest prediction of ADAS-Cog scores (R^2^ = 0.261; *p* < 0.0001). A combination of ^18^F-FDG uptake with other CSF- and PET- biomarkers further improved predictive power (^18^F-FDG + Amyloid PET: R^2^ = 0.336; *p* < 0.0001; ^18^F-FDG + Tau PET: R^2^ = 0.277; *p* < 0.0001; ^18^F-FDG + CSF Aβ42: R^2^ = 0.275; *p* < 0.0001; ^18^F-FDG + CSF t-tau: R^2^ = 0.271; *p* < 0.0001; ^18^F-FDG + CSF p-tau: R^2^ = 0.32; *p* < 0.0001), while the combination of ^18^F-FDG + p-tau/Aβ42 Ratio (R^2^ = 0.37; *p* < 0.0001) showed the highest predictive power overall.

**Table 4:** Predictive power of various biomarkers and their combinations for the alzheimer’s disease assessment Scale-Cognitive subscale (ADAS-Cog)

Biomarker	*R* ^2^	*p*
PET		
^18^F-FDG uptake PCC/Precuneus	0.17	< 0.0001
^18^F-FDG uptake parietal	0.153	< 0.0001
^18^F-FDG uptake temporal	0.217	< 0.0001
^18^F-FDG uptake frontal	0.085	< 0.0001
^18^F-FDG uptake combined regions	0.261	< 0.0001
^18^F-Florbetapir uptake	0.202	< 0.0001
^18^F-Flortaucipir uptake PCC/Precuneus	0.018	0.009
^18^F-Flortaucipir uptake parietal	0.027	0.001
^18^F-Flortaucipir uptake temporal	0.149	< 0.0001
^18^F-Flortaucipir uptake frontal	0.085	< 0.0001
^18^F-Flortaucipir uptake combined regions	0.173	< 0.0001
CSF		
CSF Aβ42	0.074	< 0.0001
t-tau	0.006	0.024
p-tau	0.146	< 0.0001
p-tau/Aβ42 Ratio	0.183	< 0.0001
Combinations		
^18^F-FDG + Amyloid PET	0.336	< 0.0001
^18^F-FDG + Tau PET	0.277	< 0.0001
Amyloid PET + Tau PET	0.207	< 0.0001
^18^F-FDG + Amyloid PET + Tau PET	0.314	< 0.0001
^18^F-FDG + CSF Aβ42	0.275	< 0.0001
^18^F-FDG + CSF t-tau	0.271	< 0.0001
^18^F-FDG + CSF p-tau	0.32	< 0.0001
^18^F-FDG + p-tau/Aβ42 Ratio	0.37	< 0.0001
^18^F-Florbetapir + CSF Aβ42	0.239	< 0.0001
^18^F-Florbetapir + CSF t-tau	0.227	< 0.0001
^18^F-Florbetapir + CSF p-tau	0.242	< 0.0001
^18^F-Florbetapir + p-tau/Aβ42 Ratio	0.25	< 0.0001
^18^F-Flortaucipir + CSF Aβ42	0.193	< 0.0001
^18^F-Flortaucipir + CSF t-tau	0.195	< 0.0001
^18^F-Flortaucipir + CSF p-tau	0.173	< 0.0001
^18^F-Flortaucipir + p-tau/Aβ42 Ratio	0.247	< 0.0001
CSF Aβ42 + t-tau	0.143	< 0.0001
CSF Aβ42 + p-tau	0.176	<0.0001
CSF Aβ42 + p-tau/Aβ42 Ratio	0.219	< 0.0001

## Discussion

With the rising prevalence of Alzheimer’s disease (AD) and the approval of the first monoclonal antibodies—aducanumab, donanemab, and lecanemab—for treating early-stage AD, an early and accurate diagnosis has become more critical than ever [10, 12, 13]. Next to the clinical assessment and neuropsychological testing, biomarkers that are able to identify different aspects of the disease in-vivo are essential for the establishment of an AD diagnosis and the identification of probable therapy responders.

According to the A/T/N model, the imaging biomarker ^18^F-FDG-PET can be classified as a marker for neuronal dysfunction providing information on the cerebral glucose metabolism in different brain regions. ^18^F-FDG-PET is able to detect patterns of cortical hypometabolism, with AD is typically characterized by a severe hypometabolism in the posterior cingulate cortex, precuneus and posterior temporal cortex [5]. However, the role of ^18^F-FDG-PET in the clinical workup of AD patients and especially its relationship to other biomarkers remains unclear. In the current study, we evaluated the diagnostic power of ^18^F-FDG-PET and its relationship to other commonly used AD biomarkers, including CSF Aβ42, t-tau, and p-tau as well as amyloid- and tau-PET results and cognition measures, using data from the ADNI cohort.

### Diagnostic Accuracy and Predictive Power

In the current study, ^18^F-FDG-PET provided the strongest power of prediction of cognitive decline and the combination of ^18^F-FDG uptake with other CSF- and PET- biomarkers further improved its predictive power, especially the combination of ^18^F-FDG-PET and the p-tau/Aβ42 ratio. Results are in line with earlier studies showing ^18^F-FDG-PET as a superior indicator of cognitive decline in AD and MCI patients compared to amyloid imaging [14, 15]. Furthermore, a study by Caminiti et al. (2018) also supported the role of statistical parametric mapping of ^18^F-FDG images and its combination with CSF Aβ42 as a strong predictor of disease progression [16].

In clinical practice, ^18^F-FDG-PET imaging plays a crucial role in detecting disease-specific patterns in individual patients, with a particular emphasis on hypometabolism in the temporal, parietal, PCC/precuneus, and frontal regions. Therefore, we wanted to focus on these brain regions in our current study rather than performing a voxel-based analysis. The metabolism in the PCC/Precuneus region showed the highest diagnostic accuracies of the four studied cortical regions for discrimination between AD and MCI or CN. Combining the FDG-uptake in all four brain regions further improved diagnostic accuracy reflecting the visual pattern. Diagnostic accuracy of ^18^F-Florbetapir PET, CSF Aβ42, p-tau, p-tau/Aβ42 ration, and regional ^18^F-Flortaucipir uptake in the PCC/Precuneus were comparable to ^18^F-FDG results. However, discrimination between MCI and CN remained challenging with all biomarkers.

The role of ^18^F-FDG-PET imaging in the diagnostic workup of suspected AD may evolve in the future, particularly with the advent of emerging anti-amyloid therapies. ^18^F-FDG-PET should be highlighted as a powerful tool, especially for detecting early-stage disease and predicting cognitive decline. Furthermore, ¹⁸F-FDG-PET provides additional diagnostic value in the differential diagnosis of differentiating between various neurodegenerative diseases based on distinct patterns of brain glucose metabolism with high sensitivity for differentiating AD from non-AD dementias [17]. However, widespread use of PET imaging is currently limited by high costs and restricted availability. To address this, a diagnostic strategy is needed that prioritizes accessible and cost-effective CSF or serum biomarkers as “first-line” tools, adding PET imaging for more complex or uncertain cases and for tracking disease progression in later stages. Furthermore, automated AI-based analysis of a specific FDG uptake score of disease-specific cortical regions might improve diagnostic accuracy in clinical routine on the individual patient level reducing inter-reader variability.

### The Relationship of ^18^F-FDG-PET to Other Biomarkers of AD

We further characterized the relationship of ^18^F-FDG-PET and other common biomarkers of AD to evaluate multimodal diagnostic approaches. A better understanding of the differences and similarities among these AD-biomarkers can support the development of more comprehensive model for AD diagnosis. Especially regarding novel antibody therapies, an accurate evaluation of AD patients in early stages of the disease and the prediction of therapy responders becomes even more important. Combining multiple biomarkers can better capture the full spectrum of pathological changes in AD by integrating different neuroimaging, laboratory, and genetic tools [18, 19]. Previous ADNI-based studies could show that the combination of structural MRI, FDG-PET and CSF increases diagnostic accuracy compared with single modalities [20, 21]. While ^18^F-FDG-PET and other PET-based biomarkers can reveal region-specific changes in the brain, fluid biomarkers offer insights into global changes in amyloid and tau pathology but lack the ability to detect region-specific cortical alterations.

### ^18^F-FDG-PET and Amyloid Biomarkers

Cortical amyloid deposition is one of the major pathological hallmarks of AD. The deposition of Aβ plaques precedes the onset of AD symptoms by more than 20 years and is suggested to affect synaptic function, neuroinflammation, synaptic destruction, and white matter alterations [22–24]. Amyloid biomarkers, including CSF and PET markers, are able to assess amyloid pathology in-vivo. Amyloid PET imaging using ^18^F-Florbetapir or other available amyloid PET tracers directly visualize the presence of cortical amyloid plaques, while CSF amyloid biomarkers detect the concentration of Aβ peptides in the CSF reflecting the production, clearance, and aggregation of Aβ peptides in the brain [25, 26].

### Amyloid PET

Whether amyloid plaque deposition leads directly to an impairment of neuronal activity and neuron loss and therefore reduced glucose metabolism in ^18^F-FDG-PET remains unclear. Our current data showed a negative correlation between regional ^18^F-FDG and ^18^F-Florbetapir uptake in all tested cortical regions. Our data is in line with earlier studies that showed an inverse correlation between regional amyloid tracer uptake and ^18^F-FDG uptake using the amyloid tracers ^18^F-Florbetapir and 11 C-PiB [27, 28]. A study by Edison et al. (2007) detected an inverse correlation of 11 C-PiB uptake and the relative regional cerebral metabolic rate of glucose in the temporal and parietal cortex in a small group of 12 AD patients and another study by Newberg et al. (2012) also showed a negative correlation of ^18^F-Florbetapir and ^18^F-FDG in the frontal, parietal, and temporal lobes in a cohort of AD patients and cognitive normal controls. Available data suggest that areas with higher amyloid burden exhibit lower glucose metabolism, indicating a link between amyloid pathology and neuronal dysfunction.

However, subgroup analysis revealed no correlation between regional ^18^F-FDG and ^18^F-Florbetapir uptake in the AD patient and CN control groups individually. In contrast, the subgroup of MCI patients showed a significant inverse correlation between ^18^F-Florbetapir and ^18^F-FDG uptake in several cortical areas. The study by Newberg et al. (2012) only detected this effect in the whole group analysis including cognitive normal subjects, but not in the analysis of the subgroup of AD patients only, which is consistent with our data on the AD patient subgroup. Therefore, the disease specific correlation might be too subtle within the AD group, potentially due to amyloid plaque deposition reaching a plateau during disease progression [29]. Amyloid plaques may have already disrupted neuronal function at earlier stages, with disease progression being driven by their presence rather than by an increase in their quantity directly correlating with neuronal impairment.

### CSF Aβ42

In an earlier study by our group, we were able demonstrate a correlation between ^18^F-Florbetaben uptake and CSF amyloid markers in a clinical cohort with memory deficits [30]. In concordance with that study, our current data also shows a correlation between ^18^F-Florbetaben and CSF Aβ42. ^18^F-Florbetaben-PET and Aβ42 seem to provide complementary information about amyloid pathology. Other recent studies were able to demonstrate that the Aβ42/40 ratio in CSF or plasma may be a more accurate predictor of amyloid-PET positivity than Aβ42 alone [14, 31, 32]. These findings indicate, that the Aβ42/40 ratio might reflect cerebral amyloid load better than Aβ42 alone. Furthermore, a combination of ^18^F-FDG-PET and Aβ42/40 ratio was proposed as the most accurate predictor of a positive amyloid scan [31].

However, while those studies have shown a correlation between ^18^F-FDG-PET and Aβ42 in predicting amyloid-PET positivity, data on the direct relationship of regional ^18^F-FDG uptake and CSF amyloid markers is very limited. In our current study, ^18^F-FDG uptake correlated with Aβ42 in several brain regions that are commonly affected by AD. A study on the indirect effect of cortical hypometabolism on the association of CSF and cognitive decline described that low baseline levels of Aβ42 were associated with low initial glucose metabolism, which is in line with our results [33]. On the contrary, one study with 34 subjects with probable AD described an unexpected inverse correlation between Aβ42 in CSF and ^18^F-FDG uptake in the precuneus/posterior cingulate assuming an increase of Aβ42 during the course of the clinical phase of AD [34]. The authors attributed these results to a possible increase in Aβ1–42 during the course of the clinical phase of AD, which parallels a decrease in glucose metabolism in key brain regions affected by AD.

However, CSF cannot provide information on region-specific cerebral amyloid load. Monitoring regional deposition of amyloid plaques in the brain is particularly relevant in the context of novel anti-amyloid therapies [35]. Given the high cost of anti-amyloid antibody treatments, amyloid PET imaging offers a valuable tool for assessing local amyloid clearance, providing insights into therapeutic effectiveness. This approach can help identify non-responders early and confirm successful treatment outcomes, thereby minimizing the risk of over-treatment.

### ^18^F-FDG-PET and Tau Biomarkers

Tau neurofibrillary tangles are another significant pathological hallmark of AD. Tau pathology is characterized by the accumulation of misfolded, insoluble tau protein aggregates in the brain affecting neuronal function, synapse loss, and neurodegeneration [36].

### Tau-PET

^18^F-Flortaucipir-PET is approved by the U.S. Food and Drug Administration for the evaluation of patients with suspected AD [37]. Tracer uptake correlates with Braak staging and regional tau burden in AD, but also shows off-target in vivo retention in basal ganglia and choroid plexus that might limit its use in non-AD tauopathies [38, 39]. We were able to demonstrate increased tau tracer deposition and hypometabolism in commonly affected brain regions of AD. However, ^18^F-FDG uptake did not correlate with ^18^F-Flortaucipir in the tested brain regions. These findings are in contrast to previous studies on tau PET imaging with ^18^F-Flortaucipir in AD patients [40–42]. Bishof et al. showed a positive correlation of tau deposition and hypometabolism in the temporal, parietal, and frontal cortex in AD patients using z-scores of FDG uptake in comparison to normal controls. Our findings might be explained by differences in the PET analysis and the low number of AD patients (*n* = 9) that underwent ^18^F-Flortaucipir-PET in our cohort, as most scans were from CN and MCI subjects, where tau accumulation and hypometabolism may still be at relatively early or spatially divergent stages. FDG-PET and tau PET reflect distinct aspects of neurodegeneration—glucose metabolism and aggregated tau—whose relationship may vary by disease stage. All reported associations are cross-sectional and likely influenced by group differences and do not imply causality. Future studies stratifying by disease stage or using longitudinal designs are needed to clarify stage-dependent biomarker interactions.

### CSF

Even though there is evidence of a direct association of tau deposition and neuronal dysfunction, data on the relationship of regional ^18^F-FDG uptake and CSF tau markers is very limited [43, 44]. In our current study, ^18^F-FDG uptake correlated inversely with both forms of CSF tau, t-tau and p-tau, in several brain regions. A study by Leuzy et al. (2019) also showed a negative association between ^18^F-FDG SUV and CSF tau levels. Results are in line with our data suggesting a direct relationship between tau and synaptic integrity and neurodegeneration [45]. As mentioned above, we found strong correlations between FDG-PET and CSF tau biomarkers, but no significant association between FDG uptake and tau PET as observed correlations may reflect group-level differences across diagnostic categories, rather than within-group relationships limited by the low number of AD patients that underwent 18F-Flortaucipir-PET in our cohort restricting the range of tau pathology. ^18^F-Flortaucipir uptake correlated with amyloid load in all tested brain regions, which is consistent with earlier studies that were able to show an association of tau pathology and amyloid plaque accumulation [6, 46–49]. Findings in early stages of the disease showed local amyloid PET tracer uptake as a strong predictor of tau PET positivity [6].

Overall, the role of ^18^F-Flortaucipir-PET in the diagnostic workup of AD patients remains unclear, especially in terms of the costs compared to CSF.

### ^18^F-FDG and Cognitive Decline

Various studies demonstrated the correlation of cognition tests, such as MMSE and ADAS-Cog, with cerebral ^18^F-FDG uptake, indicating a direct relationship between cognitive function and cerebral glucose metabolism [27, 50, 51]. These findings are in line with our results that also show a correlation of MMSE and ADAS-Cog scores with regional ^18^F-FDG uptake in the studied brain regions.

Furthermore, ^18^F-Florbetapir uptake also correlated with MMSE and ADAS-Cog in our current cohort of AD patients. These findings are in line with earlier studies using different amyloid tracers that showed a relationship between amyloid load and cognition tests [52–54]. However, other studies were not able to demonstrate these findings [52, 55]. Furthermore, histological studies could not demonstrate a relationship between amyloid plaque density and cognitive impairment [56, 57], either, suggesting that cognitive decline might not be linked directly to amyloid burden.

Regarding CSF biomarkers, only Aβ42 correlated with MMSE in the current study. Findings are in line with data from an earlier study of our group showing a correlation between Aβ42 in a clinical cohort of patients with memory deficits [30]. However, available data does not provide conclusive evidence of a direct linear relationship between Aβ42 and MMSE scores. While some studies showed a correlation between CSF markers and cognition tests assessing verbal and visuospatial episodic memory, other studies described that CSF markers were not related to the degree of cognitive impairment in AD [58–60].

### Limitations

Limitations of our study include its retrospective design. Even though the ADNI protocols are highly standardized, not all necessary data were available for every subject. As a result, several analyses were conducted on subsets of the total sample, depending on the availability of FDG-PET, amyloid PET, tau PET, and CSF measurements. While we clearly report sample sizes for each analysis, this variability introduces potential selection bias and may limit the generalizability of some findings. Furthermore, the commonly used Aβ42/40 ratio was unavailable and patients’ diagnoses were based on clinical data, and histopathological confirmation was not available.

Another limitation of this study is that biomarker correlations were analyzed across the entire cohort without stratifying by disease stage. Since biomarker relationships in Alzheimer’s disease can vary substantially along the disease continuum, especially between preclinical, prodromal, and dementia stages, our findings may reflect mixed effects rather than stage-specific patterns. Future studies should address this using stage-stratified or longitudinal analyses to better capture dynamic biomarker interactions.

Another notable limitation concerns the generalizability of our results. The ADNI cohort mainly includes well-educated, white individuals, with underrepresentation of lower education levels, lower socioeconomic status, and those residing in rural areas [61, 62]. As a result, the cohort may not adequately reflect the demographic and socioeconomic diversity of the broader population, which could limit the relevance of findings across all clinical contexts. While ADNI4 has introduced initiatives to improve diversity and inclusion, these efforts are ongoing and not yet reflected in the current dataset [63].

Overall, various fluid and imaging biomarkers of AD provide important information on AD-pathologies. Understanding their respective strengths and differences highlights the importance of a multimodal approach to comprehensively capture the spectrum of pathological changes in AD by integrating different neuroimaging, laboratory, and genetic markers [18, 19]. ^18^F-FDG-PET provides information on regional neuronal function and shows direct links to other PET and CSF biomarkers, showing a particularly strong predictive value for disease severity. Therefore, ^18^F-FDG-PET should be considered a central tool in AD diagnosis, especially in a multimodal approach in combination with CSF Aβ42 or the p-tau/Aβ42 ratio.

However, CSF biomarkers require a lumbar puncture, an invasive procedure that may not be acceptable to all patients [64, 65]. The suitability of invasive methods as lumbar puncture for CSF collection and the use of imaging modalities should be carefully considered on a patient-specific basis. Furthermore, emerging blood-based amyloid and tau measurements, such as the recently FDA-approved pTau217/β-amyloid 1–42 plasma ratio measured via the Lumipulse G platform, offer a promising non-invasive alternative and should be explored in future studies as these measurement techniques continue to improve [66–69]. Looking ahead, integrating ¹⁸F-FDG-PET with validated blood-based biomarkers could support a multimodal diagnostic approach that enhance diagnostic precision.

Biomarkers also play a crucial role in building larger datasets for big data analyses, enabling studies with larger cohorts. This can enhance information gain and support the creation of databases for deep learning strategies, optimizing a multi-biomarker approach tailored to individual patients. Furthermore, as ^18^F-FDG-PET does provide crucial information on the state of impaired brain areas, it can not only identify AD patients, but also other forms of dementia that might show overlapping clinical symptoms or biomarkers.

## Conclusion

Biomarkers are essential for the diagnosis of AD reflecting different aspects of AD pathology. ^18^F-FDG-PET, as an independent biomarker of neurodegeneration, can directly detect regional changes in neuronal function and is a powerful tool to identify AD patients and predict disease severity. A combination with fluid biomarkers appears to be the most accurate approach for the characterization of AD, ensuring a more comprehensive understanding of the disease.

## Supplementary Information

Below is the link to the electronic supplementary material.

ESM 1 (DOCX 3.56 MB)

## Data Availability

The datasets used during the current study are available from the corresponding author upon request.

## Declarations

### Preprint Sharing

This manuscript has not been previously published as a preprint.
