# Supplementary material for: 18F-FDG-PET and Multimodal Biomarker Integration: A Powerful Tool for Alzheimer’s Disease Diagnosis
Source: Nucl Med Mol Imaging. 2025 Jul 10;59(6):453–71. doi: 10.1007/s13139-025-00932-2 (PMC12669464; doi:10.1007/s13139-025-00932-2)
Supplement: Supplementary file 1 — (DOCX 3.56 MB) [file 13139_2025_932_MOESM1_ESM.docx]

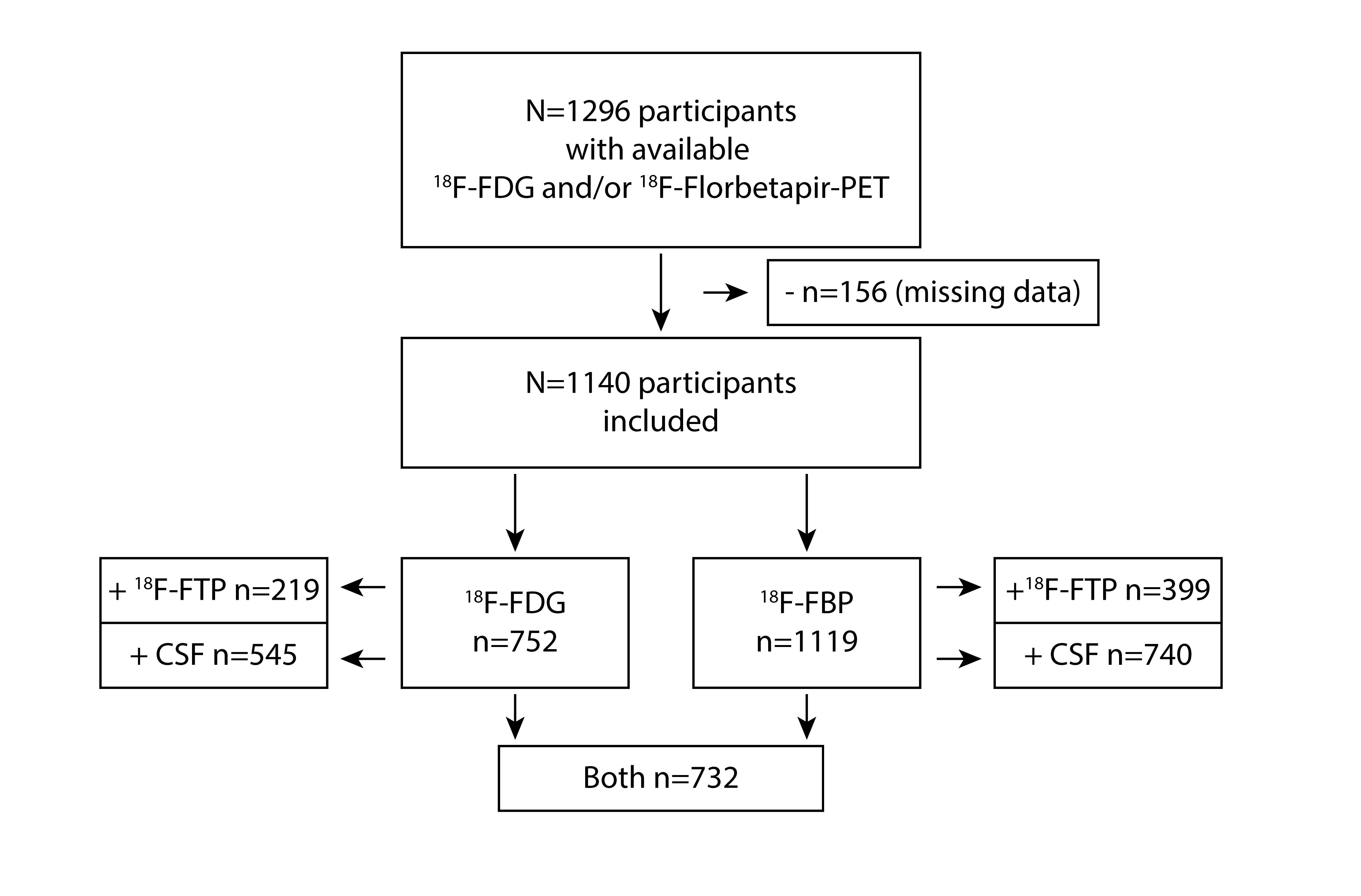


**Supplementary Figure 1.** Flowchart illustrating patient inclusion and availability of PET imaging. Of the 1296 participants initially assessed, 1140 were included. Among these, 1119 underwent 18F-Florbetaben (FBP) PET and 752 underwent 18F-FDG PET. A total of 732 participants received both PET scans.
